# Age-related reduction and independent predictors of toe flexor strength in middle-aged men

**DOI:** 10.1186/s13047-017-0196-3

**Published:** 2017-03-27

**Authors:** Masataka Suwa, Takayuki Imoto, Akira Kida, Mitsunori Iwase, Takashi Yokochi

**Affiliations:** 1 Health Support Center WELPO, Toyota Motor Corporation, 1-1, Ipponmatsu, Iwakura-cho, Toyota, Aichi 444-2225 Japan; 2 Toyota Memorial Hospital, 1-1, Heiwa-cho, Toyota, Aichi 471-8513 Japan

**Keywords:** Aging, Blood pressure, Handgrip, Hyperglycemia, Skeletal muscle, Toe flexion

## Abstract

**Background:**

Toe flexor muscles play an important role in posture and locomotion, and poor toe flexor strength is a risk factor for falls. In this cross-sectional study, we estimated the age-related change in toe flexor strength and compared it with that of handgrip strength. Independent factors predicting toe flexor and handgrip strength were also determined.

**Methods:**

A total of 1401 male (aged 35–59 years) study participants were divided into five groups according to their chronological age; 35–39, 40–44, 45–49, 50–54, and 55–59 years. Toe flexor and handgrip strength, anthropometry, and resting blood pressure were measured. Fasting blood samples were collected to measure blood glucose, triglycerides, high- and low-density lipoprotein-cholesterols, and albumin. A self-administered lifestyle questionnaire was conducted.

**Results:**

Decline in absolute toe flexor and handgrip strength began in the age groups 50–55 and 55–59 years, respectively. In comparison to the mean values of the youngest group, relative toe flexor strength (87.0 ± 26.6%) was significantly lower than handgrip strength (94.4 ± 13.1%) for the oldest group. Multiple regression analyses showed that independent factors predicting both toe flexor and handgrip strength were lean body mass, age, serum albumin, drinking habit, and fat mass. Additionally, fasting blood glucose, diastolic blood pressure, sleeping time and exercise habit were predicting factors of toe flexor strength but not of handgrip strength.

**Conclusions:**

Age-related reduction in toe flexor strength was earlier and greater than handgrip strength, and toe flexor strength reflects body composition and metabolic status.

## Background

The foot is the only part of the human body that comes into contact with the ground during gait and upright stance, and it can be speculated that the toe plays an important role in stabilizing posture during standing and locomotive activities, such as walking and running [1]. Toe flexion is an action produced by activity of plantar intrinsic (flexor hallucis brevis, flexor digitorum brevis, and lumbricals pedis) and extrinsic (flexor hallucis longus and flexor digitorum longus) muscles [1]. Toe flexor muscles are associated with arch support [2], posture stability [3], locomotive activity [4–6], jump performance [7, 8], and amount of physical activity [9]. Moreover, poor toe flexor strength is a risk factor for falls in the older individuals [10, 11]. Toe flexor strength is positively correlated with knee extensor strength [12]. Toe flexor strength measurement is assumed to be a quick, easy, and inexpensive way to estimate lower limb muscle strength. Thus, measurement of toe flexor strength would be useful in monitoring physical functions and activities of daily living. However, little is known regarding the factors associated with toe flexor strength.

Skeletal muscle functions and volume reduce with age [13, 14], and the rate of such reductions differ between regions and muscles. Skeletal muscle strength and volume of the lower body decrease faster than those of the upper body [15–17]. The rate of the age-related reduction seen differ between flexor and extensor muscle strength and volume [18, 19]. In addition, the pattern of age-related reduction in muscle force varies considerably [16, 20].

Toe flexor strength also decreases with age [3, 21]. The percentage difference in toe flexor strength between older men aged 65–88 years and young men aged 18–23 years (55.2% of young men) is lower than that for handgrip strength (79.2% of young men), suggesting that loss of toe flexor strength occurs earlier compared with loss of handgrip strength [12]. However, the pattern of reduction during middle age remains unclear.

Poor skeletal muscle strength increases the risk of death and several diseases in the middle-aged and older individuals. Handgrip strength has been measured in many epidemiological studies showing that poor handgrip strength is associated with all-cause and cardiovascular mortality [22–24], heart disease [23, 24], stroke [23, 24], diabetes mellitus [25, 26], hypertension [25], metabolic syndrome [27], cognitive decline [28], and cancer [23]. However, handgrip strength does not relate to physical activity level, whereas lower limb muscle strength, including toe flexor and knee extensor, significantly reflect it [21, 29]. Low physical activity level is another risk factor for death and several health-related problems [30]. Taken together, it is likely that toe flexor strength is a better marker of several diseases than handgrip strength.

Based on this information, we designed this study to clarify the essential properties such as age-related change and predictors of toe flexor strength. In the present cross-sectional study we estimated and compared age-related changes in toe flexor and handgrip strength in middle-aged men. We further investigated which factors concerning medical, anthropometrical, and life style-related parameters predicted toe flexor and handgrip strength.

## Methods

### Study design and population

The present study was performed as part of the baseline survey of the Toyota Motor Corporation Physical Activity and Fitness Study (TMCPAFS), conducted from October 2015 to January 2016. Participants in the baseline study were 1410 Japanese male employees, aged 35–59 years of the Toyota Motor Corporation, Toyota, Aichi, Japan. Nine individuals were excluded because of incomplete data; thus, 1401 participants were included in the current study. All participants received annual medical examinations in accordance with the Industrial Safety and Health Law of Japan. Employees were required by law to participate and all clinical data were supplied as medical examination data. The study was conducted in accordance with the Declaration of Helsinki. The study protocol was approved by the Ethics Committee in the Toyota Memorial Hospital. All of the subjects provided consent for participation in this study.

### Medical examinations

After an overnight fast of at least 11 h, subjects underwent examinations including anthropometry, resting systolic and diastolic blood pressures (SBP and DBP, respectively) measurements, and blood chemistry analyses. Height, body mass (BM) and percentage of body fat (%fat), determined by bioelectrical impedance, were measured using an automated measuring instrument (BF-220; Tanita, Tokyo, Japan). Fat mass (FM) was calculated as the multiplication of BM by %fat/100, and lean body mass (LBM) was calculated as the subtraction of FM from BM. Body mass index (BMI) and lean BMI (LBMI) were calculated as BM and LBM divided by height squared, respectively. Waist circumference (WC) was measured at the level of the umbilicus in a standing position while breathing normally (at the end of expiration while breathing gently). Fasting blood samples were drawn from seated participants from the antecubital vein. Fasting blood glucose (FBG) was measured by the hexokinase-glucose-6-phosphate dehydrogenase method (Eiken Chemical Co, Ltd, Tokyo, Japan). Concentrations of triglycerides (TG) were measured by enzymatic colorimetric analysis (JSCC and ReCCS standard method). High- and low-density lipoprotein-cholesterols (HDL-C and LDL-C, respectively) were measured with the chemically modified enzyme method (Metaboredo®HDL-C and metaboredo®LDL-C; Kyowa Medex Co, Ltd, Tokyo, Japan). Total proteins were measured in accordance with a burette method. Serum albumin was assayed with modified bromocresol purple method. In addition, a self-administered questionnaire was conducted to assess level of 30 min or more of exercise (none, 1; 1 time/week, 2; 2–6 times/week, 3; every day, 4), smoking (never, 1; former, 2; current, 3) and alcohol (none, 1; sometimes, 2; ~3 times/week, 3; every day, 4) habits and sleeping time.

### Measurements of muscle strength

**Fig. 1:**
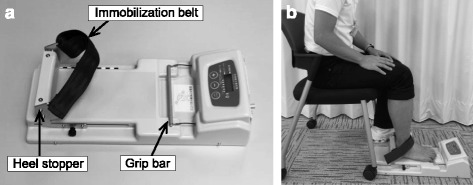
Measurement of toe flexor strength. **a** the toe grip dynamometer and parts of the instrument. **b** test of toe flexor strength using a toe grip dynamometer. The subject sits on a chair with their trunk in the vertical position and hip and knee joints flexed at approximately 90°. One foot is placed on the dynamometer, and the first proximal phalanx of the foot is positioned on a grip bar with the heel position fixed using a heel stopper and immobilization belt

Toe flexor strength was measured using a toe grip dynamometer (T.K.K. 3364, Takei Scientific Instruments Co., Ltd, Niigata, Japan) (Fig. 1a). The intraclass correlation coefficients (ICCs) of this apparatus in middle-aged men (40–59 years) are previously described, and ICC (1.1), as an intrarater reliability of single measure, and ICC (2.1), as an interrater reliability of single measure, are 0.88 and 0.97, respectively [31]. The ICC scores from 0.8 to 1.0 are almost perfect agreement [32]. Measurement of toe flexor strength is illustrated in Fig. 1b. Subjects sit on a chair with their trunk in the vertical position and hip and knee joints flexed at approximately 90° with the ankle joint in a plantargrade (at approximately 90°). The first proximal phalanx of the foot is positioned on a grip bar and heel position is fixed using a heel stopper and immobilization belt (Fig. 1b). After sufficient training trials, toe flexor strength is measured twice. Measurements were performed on both right and left toes, and the mean maximum force of each toe was used in subsequent analyses.

Handgrip strength was measured using a handgrip dynamometer (T.K.K. 5401; Takei Scientific Instruments). Measurements were made in duplicate in each hand, and the mean maximum force of each hand was used in analyses.

### Statistical analysis

Data are expressed as mean ± standard deviation (SD). Participants were divided into five groups according to their chronological age, 35–39, 40–44, 45–49, 50–54, and 55–59 years, to analyze age-related changes in toe flexor and handgrip strength. One-way analysis of variance (ANOVA) was used to compare mean values between the five groups. The significance of between-group differences was determined using Tukey post hoc testing if the overall ANOVA was significant. Lifestyle differences between the five groups, including exercise (none, or more than one times/week), and drinking (none, or sometimes and more) and smoking (never, former, or current) habits, were compared using a chi-square test. The significance of between-group differences was also determined by chi-square test with sequentially rejective Bonferroni correction of significance levels. To compare relative changes from the mean values of the youngest age group between toe flexor and handgrip strength, two-way ANOVA (toe flexor or handgrip × age group) was used. Additionally, to compare relative values of muscle strength from the mean values of the youngest group between toe flexor and handgrip strength in each age group, we used a paired t–test. To predict the factors associated with toe flexor and handgrip strength, univariate correlation analyses and stepwise multiple regression analyses were performed. Variables for the stepwise linear regression model were selected on the basis of correlation analyses and included variables considered to be related to muscle strength [25, 33–38]. Height, BM and WC were not included in multiple regression to analyze the effects of adipose tissue and other tissues separately and also because they were highly correlated (r ≥ 0.60) with LBM and/or FM.

Differences were considered significant when *P* < 0.05. SPSS software (Version 23.0 for Windows, SPSS, Inc., Chicago, IL, USA) was used for all statistical analyses.

## Results

### Characteristics of the participants

Table 1 shows participant characteristics according to age group, including anthropometry, medical examination data, and lifestyle. ANOVA or chi-square test indicated significant differences between the groups (*P* < 0.05), with the following exceptions: %fat, FM, HDL-C, total protein, drinking habit, or current smoking.

**Table 1:** Characteristics of the study participants by age group

	Total	35–39 years	40–44 years	45–49 years	50–54 years	55–59 years	P^a^
N =	1401	315	198	140	343	405	
Age (years)	48.0 ± 8.1	36.0 ± 1.4	42.3 ± 1.8*	47.5 ± 0.5*†	51.6 ± 0.5*†‡	57.2 ± 1.8*†‡§	< 0.001
Height (cm)	170.5 ± 5.9	171.5 ± 5.7	172.4 ± 5.5	171.3 ± 5.3	170.0 ± 6.0*†	168.9 ± 5.9*†‡	< 0.001
BM (kg)	67.9 ± 10.1	67.2 ± 10.2	70.0 ± 10.2*	68.9 ± 8.9	67.8 ± 9.9	67.3 ± 10.5†	0.012
%fat (%)	22.4 ± 5.6	22.1 ± 5.8	22.7 ± 5.5	22.6 ± 4.7	23.0 ± 5.9	22.1 ± 5.4	0.162
LBM (kg)	52.3 ± 5.5	51.9 ± 5.1	53.7 ± 5.4*	53.1 ± 5.0	51.9 ± 5.5†	52.0 ± 5.8†	< 0.001
FM (kg)	11.8 ± 3.6	11.5 ± 3.7	12.3 ± 3.6	12.0 ± 3.1	12.0 ± 3.6	11.6 ± 3.5	0.101
BMI (kg/m^2^)	23.4 ± 3.3	22.8 ± 3.3	23.5 ± 3.3	23.5 ± 2.6	23.5 ± 3.2	23.6 ± 3.4	0.018
LBMI (kg/m^2^)	18.0 ± 1.5	17.6 ± 1.5	18.0 ± 1.6*	18.1 ± 1.2*	17.9 ± 1.6	18.2 ± 1.6*	< 0.001
WC (cm)	81.9 ± 8.9	79.3 ± 8.5	81.7 ± 9.2*	81.5 ± 7.6*	82.6 ± 8.7*	83.4 ± 9.1*	< 0.001
FBG (mg/dL)	99.4 ± 11.5	93.7 ± 11.5	96.6 ± 11.5	98.8 ± 19.4*†	100.1 ± 15.8*†	104.8 ± 17.6*†‡§	< 0.001
SBP (mmHg)	118.4 ± 13.8	114.5 ± 11.3	115.4 ± 12.8	116.7 ± 13.6	119.7 ± 13.6*†	122.3 ± 15.2*†‡	< 0.001
DBP (mmHg)	76.9 ± 9.3	73.8 ± 8.2	75.7 ± 8.4	77.2 ± 9.1	78.1 ± 9.6*†	78.8 ± 9.6*†	< 0.001
TG (mg/dL)	116.7 ± 88.1	100.7 ± 68.4	110.5 ± 84.4	131.1 ± 111.7*	120.1 ± 77.5	124.3 ± 100.3*	0.001
HDL-C (mg/dL)	60.6 ± 16.2	60.8 ± 15.2	61.3 ± 16.3	59.2 ± 15.5	59.7 ± 16.5	61.4 ± 16.7	0.487
LDL-C m (g/dL)	126.4 ± 29.9	122.2 ± 31.5	125.3 ± 29.3	130.0 ± 31.2*	129.7 ± 28.8*	126.2 ± 28.9	0.012
Total protein (g/dL)	7.12 ± 0.36	7.14 ± 0.34	7.11 ± 0.33	7.13 ± 0.36	7.09 ± 0.37	7.12 ± 0.36	0.538
Albumin (g/dL)	4.51 ± 0.25	4.59 ± 0.24	4.53 ± 0.23	4.53 ± 0.27*	4.49 ± 0.24*	4.46 ± 0.26*†‡	< 0.001
Sleeping time (h/day)	6.21 ± 0.93	6.18 ± 0.97	6.10 ± 1.00	6.08 ± 0.77	6.21 ± 0.90	6.32 ± 0.94‡	0.021
Exercise habit (%)^b,c^	67.5	58.1	59.6	74.3*†	70.8*†	73.6*†	< 0.001
Drinkers (%)^b^	76.8	76.2	75.3	79.3	74.6	79.0	0.586
Current smokers (%)^b^	38.5	41.9	35.9	30.7	42.3	36.5	0.077
Former smokers (%)^b^	15.2	8.6	13.1	17.1*	19.5*	17.0*	0.001
Never smokers (%)^b^	46.3	49.5	51.0	52.1	38.1*†‡	46.4§	0.007

Data are mean ± SD. ^a^ *P* values from ANOVA or chi-square test. ^b^ Chi-square test. ^c^ At least one period of exercise per week. *, †, ‡, and §, significantly different from the groups aged 35–39, 40–44, 45–49, and 50–54 years, respectively (*P* < 0.05)

### Muscle strength according to age

Table 2 shows the absolute values and relative values to BM or LBM of toe flexor and handgrip strength. Both toe flexor and handgrip strength were reduced with age. Reduction of the absolute toe flexor strength occurred first in the group aged 50–54 years, whereas a reduction in handgrip strength occurred first in the group aged 55–59 years. Reductions in toe flexor and handgrip strength relative to BM or LBM were both seen first in the group aged 40–44 years, and reductions in both strengths relative to LBM further proceeded in the group aged 55–59 years. Correlation coefficients between age and toe flexor strength, toe flexor strength/BM, toe flexor strength/LBM, handgrip strength, handgrip strength/BM, and handgrip strength/LBM were *r* = –0.185, –0.167, –0.174, –0.152, –0.111, and –0.129, respectively (*P* < 0.001). Correlation coefficients between toe flexor and handgrip strength in absolute values and values relative to BM and LBM were *r* = 0.339, 0.415, and 0.309 (*P* < 0.001), respectively.

**Table 2:** Toe flexor and handgrip strength by age group

	Total	35–39 years	40–44 years	45–49 years	50–54 years	55–59 years	P^a^
N =	1401	315	198	140	343	405	
Toe flexor strength (kg)	20.1 ± 6.0	21.8 ± 5.9	20.7 ± 6.3	21.0 ± 6.1	19.3 ± 5.8*‡	18.9 ± 5.8*†‡	< 0.001
Toe flexor strength/BM (kg/kg)	0.300 ± 0.094	0.329 ± 0.095	0.300 ± 0.093*	0.307 ± 0.091	0.288 ± 0.089*	0.287 ± 0.095*	< 0.001
Toe flexor strength/LBM (kg/kg)	0.386 ± 0.115	0.421 ± 0.114	0.386 ± 0.114*	0.396 ± 0.113	0.374 ± 0.110*	0.367 ± 0.114*‡	< 0.001
Handgrip strength (kg)	41.2 ± 5.7	42.1 ± 5.6	42.0 ± 5.6	41.4 ± 5.7	41.5 ± 5.7	39.8 ± 5.5*†‡§	< 0.001
Handgrip strength/BM (kg/kg)	0.616 ± 0.101	0.637 ± 0.103	0.609 ± 0.100*	0.606 ± 0.084*	0.620 ± 0.97	0.601 ± 0.103*	< 0.001
Handgrip strength/LBM (kg/kg)	0.792 ± 0.107	0.816 ± 0.106	0.786 ± 0.106*	0.782 ± 0.100*	0.803 ± 0.102	0.770 ± 0.109*§	< 0.001

Data are mean ± SD. ^a^ *P* values from ANOVA. *, †, ‡, and §, significantly different from the groups aged 35–39, 40–44, 45–49, and 50–54 years, respectively (*P* < 0.05)

**Fig. 2:**
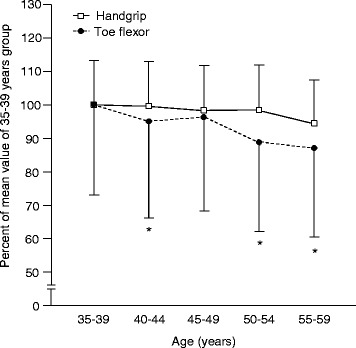
Relative differences between toe flexor and handgrip strength from the youngest group. Data are presented as mean ± SD. * *P* < 0.05 vs. handgrip strength

Figure 2 indicates the relative differences of toe flexor and handgrip strength from the youngest age group. Both age and muscle strength type effects indicated significance by two-way factorial ANOVA (*P* < 0.001). Additionally, an interaction effect was also observed (*P* < 0.001). Relative values of toe flexor strength in the groups aged 40–44, 50–54, and 55–59 years were significantly lower than those of handgrip strength. Relative toe flexor and handgrip strength in the group 55–59 years were 87.0 ± 26.6% and 94.4 ± 13.1% of those in the group 35–39 years, respectively. Similar reduction patterns were observed in the relative muscle strength/BM and muscle strength/LBM data from the youngest group (data not shown).

### Factors predicting muscle strength

The findings of univariate regression analysis indicated that the absolute value of toe flexor strength was positively associated with height, BM, LBM, FM, BMI, LBMI, and albumin and negatively associated with age, FBG, and sleeping time. The absolute value of handgrip strength was positively associated with height, BM, LBM, FM, BMI, LBMI, WC, and albumin and negatively associated with age and sleeping time. We then performed stepwise multiple-regression analyses (Table 3). Independent positive factors predicting toe flexor strength were LBM, albumin, drinking habit, DBP, and exercise habit and negative factors were age, FBG, FM, and sleeping time. In contrast, TG, HDL-C, LDL-C or smoking habit did not affect the multiple regression. Independent positive factors of handgrip strength were LBM, albumin, and drinking habit and negative factors were age and FM. However, TG, HDL-C, LDL-C, exercise habit, sleeping time, or smoking habit did not affect the multiple regression in handgrip strength.

**Table 3:** Stepwise multiple-regression analyses of factors affecting toe flexor and handgrip strength

	Regression coefficient	SE	β	P	R^2^ change (%)
Toe flexor strength^a^					
Constant	1.967	3.889		0.613	
LBM	0.277	0.036	0.252	< 0.001	4.1
Age	−0.128	0.021	−0.173	< 0.001	3.2
Albumin	2.328	0.629	0.097	< 0.001	0.8
Drinking habit	0.364	0.132	0.072	0.006	0.5
FBG	−0.026	0.011	−0.067	0.016	0.5
DBP	0.047	0.018	0.073	0.008	0.3
FM	−0.145	0.057	−0.086	0.011	0.4
Sleeping time	−0.342	0.165	−0.053	0.038	0.3
Exercise habit	0.682	0.330	0.053	0.039	0.3
Handgrip strength^b^					
Constant	11.486	3.243		0.225	
LBM	0.474	0.032	0.458	< 0.001	15.1
Age	−0.092	0.017	−0.133	< 0.001	2.0
Albumin	2.301	0.559	0.102	< 0.001	0.8
FM	−0.176	0.049	−0.111	< 0.001	0.8
Drinking habit	0.397	0.117	0.083	0.001	0.7

*SE* Standard error

^a^ Excluded variables: TG, LDL-C, HDL-C and smoking habit. R^2^ = 0.104, adjusted R^2^ = 0.098, *P* < 0.001

^b^ Excluded variables: TG, LDL-C, HDL-C, exercise habit, sleeping time, smoking habit, DBP, and FBG. R^2^ = 0.193, adjusted R^2^ = 0.190, *P* < 0.001

## Discussion

To our knowledge, this is the first study to evaluate age-related changes in toe flexor strength in detail by comparing them with changes in handgrip strength in middle age. The current cross-sectional study showed different patterns and rates of age-related changes between toe flexor and handgrip strength. Decline of absolute toe flexor and handgrip strength began in subjects aged 50–55 years and 55–59 years, respectively, suggesting that the age-related decline in toe flexor strength initiates earlier than that for handgrip strength. Reductions in both toe flexor and handgrip strength relative to BM or LBM were observed in the group aged 40–44 years. The reductions of both strength measures relative to LBM further proceeded in the group aged 55–59 years. Moreover, the reduction rate of toe flexor strength was greater than that of handgrip strength.

Stepwise multiple-regression analyses indicated that LBM, albumin, FM, and drinking habit were independent factors predicting both toe flexor and handgrip strength as well as age. Moreover, FBG, DBP, FM, sleeping time, and exercise habit were further independent factors of toe flexor strength. Such different predicting factors between toe flexor and handgrip strength might at least, in part, explain the different pattern of age-related decline between toe flexor and handgrip strength. Handgrip strength is considered a good marker of physical health, and has been measured in many epidemiological studies which predicted mortality and several diseases and dysfunctions specifically observed in middle-aged and older persons [22–24, 26, 28]. The observations in the current study might imply that toe flexor strength is a better marker of age-related decline of physical health compared with handgrip strength. Further studies are required to investigate whether toe flexor strength reflects and/or predicts age-related disorders.

A previous study demonstrated that reduction in absolute toe flexor strength began in men in their 50s [21]. This result is consistent with the current study results, as we also showed that toe flexor strength began to decline in participants aged in their early 50s. However, we showed a significant age-related decline in toe flexor strength/BM during late 30s to 50s. Uritani et al. [21] did not indicate such changes during their ages. One possible explanation for such inconsistent results is the different number of participants in each group between the study of Uritani et al. (*n* = 72–101) and this study (*n* = 140–405).

Another previous study indicated that poor muscular strength quantified by a combination of leg and bench presses was a risk factor for metabolic syndrome [39]. Previous cross-sectional observations have also shown that lower muscle strength or mass are associated with insulin resistance, dyslipidemia, type 2 diabetes, and elevated blood pressure [27, 40, 41]. Furthermore, age-related loss of muscle strength and mass are accelerated in patients with type 2 diabetes in compared with nondiabetic counterparts [42]. Taken together, poor muscle strength can be both a cause and a result of insulin resistance-related metabolic diseases. Consistent with these results, the present study showed that FBG and DBP were independent predicting factors of toe flexor strength. Conversely, FBG or DBP were not a predicting factor of handgrip strength. Therefore, it is likely that weak toe flexor strength is a better marker of metabolic abnormalities than handgrip strength.

The physiological or biochemical mechanisms underlying the associations between toe flexor strength and FBG are unknown. One possibility is that poor toe flexor strength is associated with impaired metabolic function in skeletal muscle cells. Because skeletal muscle plays an important role in regulating the whole body glucose and lipid metabolism via insulin- and contraction-induced signals [43], it is intuitive that impaired skeletal muscle functions lead to chronic metabolic abnormalities. Another possibility is that such associations are linked to the physical activity level and/or gait speed. Toe flexor strength reflects the level of physical activity [9, 12] and gait speed [5]. Physical activity intervention reduces the risk of type 2 diabetes and hypertension [44]. Gait speed is slower in patients with diabetes than non-diabetic subjects [40]. Therefore, it is likely that physical activity level and gait speed have an impact on the association between toe flexor strength and FBG observed in this study. A further possibility is that accumulating glycated myofibrillar proteins owing to elevated glucose levels reduce muscle fiber properties. For example, glycation of myosin protein reduces actin motility and adenosine triphosphatase activity [45], which would decrease the force production capacity of muscle fiber. Additionally, toe flexor strength in type 2 diabetic patients with polyneuropathy is lower than in those without polyneuropathy [46]. Thus, it is possible that diabetic neuropathy is also involved.

The current study showed that both toe flexor and handgrip strength were positively associated with serum albumin levels. Contrasting results have been reported in previous studies regarding albumin levels and muscle strength. Consistent with our results, previous cross-sectional data also show that serum albumin levels are associated with handgrip strength and leg power in older men and women [47, 48]. In contrast, serum albumin level is not associated with handgrip strength in young and old men and women [49]. In longitudinal studies, lower serum albumin levels were associated with future reduction in muscle mass [50] and handgrip strength [47] in older men and women. Furthermore, another study demonstrated that the serum albumin level does not predict changes in handgrip strength and leg power in older men [48]. We do not have any appropriate explanations for such inconsistent results between the previous studies and this study. However, the participants in this study were Japanese middle-aged (35–59 years) men while the participants in the previous studies were young (aged 18–30 years) or older (aged ≥65 year). Moreover, all of the participants in our study were employees of one corporation. It is possible that such a relatively low heterogeneous population contributed to the observed statistically significant association between serum albumin level and muscle strength in this study.

Serum albumin level is an indicator of nutritional status [51]. A low albumin level is associated with malnutrition [52] and low animal protein intake [53]. Albumin possesses an anti-oxidant capacity [54], which protects muscle tissue from oxidative damage, one of the causes of muscle atrophy and impaired force generation [45]. Serum albumin level is a possible regulator of testosterone level [55]. Albumin is an activator of phosphatidylinositol 3-kinase (PI3K) [56], which promotes muscle hypertrophy via the Akt-mammalian target of rapamycin pathway [57]. Lower serum albumin is associated with increasing inflammatory status which increases inflammatory cytokine levels [58, 59]. Inflammatory cytokines such as interleukin-6 and tumor necrosis factor-α promote muscle atrophy [57]. Interestingly, in pancreatic β cells, albumin also positively regulates the PI3K-Akt signaling pathway, which protects against cytokine-induced β-cell death [56]. It is thus likely that a lower albumin level promotes diabetes-induced muscle weakness, followed by cytokine-induced impairment of insulin secretion in pancreas, and elevation of circulating glucose levels. These mechanisms are potential candidates to account for the relationship between muscle strength and serum albumin levels. However, the cross-sectional nature of this study limits the ability to draw causal inferences from this relationship. Further, longitudinal studies are needed to refine such mechanisms and to fully understand the relationship between toe flexor strength and serum albumin.

Toe flexor strength is associated with the amount of low-to-moderate intensity physical activity [9, 12]. A previous study showed that toe flexor strength/BM in a higher daily step counts group (≥8000) was greater than that in a lower daily step counts group, however, such a difference in knee extensor strength/BM was not observed in women aged 52–78 years [9]. In addition, the study demonstrated significant partial correlation coefficients, adjusted by age, between toe flexor strength/BM and physical activity levels, including daily step counts and amount of low-to-moderate intensity physical activity (≤6 metabolic equivalents) estimated using an accelerometer; similar correlations were not observed in knee flexor strength/BM. We have also previously shown that toe flexor strength is correlated with the daily sum of standing and sitting times assessed using a self-administered questionnaire [12]. Physical activity can be further divided into exercise and non-exercise activity such as labor and household affairs, and the activities in these previous studies [9, 12] would comprise both types of activity. We herein demonstrated that exercise habit was an independent predictor of toe flexor strength. Collectively these results would imply that toe flexor strength at least partially reflects level of exercise activity. However, it remains unknown whether toe flexor strength also reflects the amount of non-exercise physical activity. In addition, this study did not estimate the intensity of exercise. Therefore, associations between toe flexor strength and intensity of exercise or total amount of exercise (intensity × time) has been still unknown.

The current study showed that both toe flexor and handgrip strength were positively related to drinking habit. A possible explanation of such relation is that alcohol consumption promotes muscle mass and strength. Alcohol intake (0.5 g/kg) increases circulating testosterone level 2 h after ingestion in men [35]. Another study using 0.675 g/kg alcohol intake also indicates increasing testosterone level [60]. In contrast, higher dose of alcohol (≥1.5 g/kg) rather decreases circulating testosterone level [61, 62]. On the basis of these results, it is likely that low to moderate dose of alcohol consumption has beneficial effects on skeletal muscle mass and functions.

In the current study, multiple regression analysis showed that coefficient of determination value of toe flexor strength was very small for many variables, speculating that other factors not estimated in this study largely account for the variability of toe flexor strength. Furthermore, standardised partial regression coefficients of albumin, drinking habit, FBG, DBP, FM, sleeping time, and exercise habit in toe flexor strength were very small, and each measurement explained less than 1% variance in toe flexor strength. The clinical importance of these small associations might be less or even questioned. Further studies concerning other factors accounting for toe flexor strength and clinical importance of valuables associated with toe flexor strength are called for.

This study has several limitations. First, the cross-sectional nature of this study limits our ability to draw causal inferences from the relationships observed, and differences between groups did not indicate longitudinal changes. Second, the characteristics of participants in this study limit the generalizability. All participants were Japanese employees in one corporation of the manufacturing industry, thus, there may be some bias. And also, all participants in this study were men. It is unknown whether age-related reduction and independent regulators of muscle strength are the same for women. A previous study showed that toe flexor strength in one’s 70s is 38.5% lower than in one’s 20s in men, but 29.8% lower in women [21]. It is likely that toe flexor strength in women is more gently reduced with age than that in men. Further studies estimating toe flexor strength in women are required. Third, this study did not consider medical history or medication use, such as hypoglycemic or hypotensive drugs, therefore, relationships between toe flexor strength and FBG and DBP might include some bias.

## Conclusions

This cross-sectional study demonstrated an age-related decline in toe flexor and handgrip strength, and the reduction rate in toe flexor strength was greater than that in handgrip strength in Japanese male workers aged 35–59 years. The LBM, age, albumin, FM, and drinking habit are independent factors predicting both toe flexor and handgrip strength, and FBG, DBP, sleeping time, and exercise habit are further independent factors of toe flexor strength. Poor toe flexor strength may be a better marker of age-related decline in physical fitness and metabolic status than handgrip strength.

## Abbreviations

%fat: Percentage of body fat
ANOVA: Analysis of variance
BM: Body mass
BMI: Body mass index
DBP: Diastolic blood pressure
FBG: Fasting blood glucose
FM: Fat mass
HDL-C: High-density lipoprotein-cholesterol
LBM: Lean body mass
LBMI: Lean body mass index
LDL-C: Low-density lipoprotein-cholesterol
PI3K: Phosphatidylinositol 3-kinase
SBP: Systolic blood pressure
SD: Standard deviation
SE: Standard error
TG: Triglyceride
WC: Waist circumference
